# Normal glutamate but elevated myo-inositol in anterior cingulate cortex in recovered depressed patients

**DOI:** 10.1016/j.jad.2009.02.022

**Published:** 2009-12

**Authors:** Matthew J. Taylor, Sudhakar Selvaraj, Ray Norbury, Peter Jezzard, Philip J. Cowen

**Affiliations:** a Department of Psychiatry, University of Oxford, Warneford Hospital, Oxford, United Kingdom; b The Centre for Functional Magnetic Resonance Imaging of the Brain, University of Oxford, John Radcliffe Hospital, Oxford, United Kingdom

**Keywords:** Magnetic resonance spectroscopy, Unipolar disorder, Glutamate, Glutamine, Myo-inositol, Frontal cortex

## Abstract

**Background:**

MRS studies of acutely depressed patients reveal decreased levels of total glutamate and glutamine (Glx) in frontal cortex which may reflect abnormalities of glutamate–glutamine cycling through astrocytes. Frontal Glx levels appear to be normalised after recovery from depression, but it is not known if this composite measure masks ongoing differences in glutamate or glutamine alone.

**Methods:**

Medication-free, fully recovered patients with a history of DSM-IV recurrent major depressive disorder (*n* = 14) and healthy controls (*n* = 16) were scanned at 3T. Short echo time PRESS and PRESS-J spectra were acquired from a 12 cm^3^ voxel of frontal cortex incorporating the anterior cingulate.

**Results:**

Levels of Glx and of glutamate alone did not differ between groups. However, myo-inositol concentrations were significantly higher in those with a history of depression than in controls.

**Limitations:**

Abnormal MRS measures were not demonstrated during episodes of depression for these participants, so any evidence of changes with recovery is indirect.

**Conclusions:**

The normal glutamatergic measures combined with elevated levels of the astrocytic marker, myo-inositol, suggest that recovery from depression may be associated with changes in glial function in frontal cortex.

## 1 Introduction

There is a growing interest in the role of brain glutamate mechanisms in depression (Sanacora et al., 2008), heightened by the observation that drugs altering glutamate activity such as the NMDA receptor antagonist, ketamine, and the glutamate release inhibitor, riluzole, may have beneficial effects in patients with treatment resistant depression (Sanacora et al., 2004; Zarate et al., 2006).

Measuring glutamate activity in the brain in humans presents challenges but the use of magnetic resonance spectroscopy (MRS) provides a useful approach. Several studies have applied proton MRS to study glutamate levels in depression and overall the evidence suggests that acutely depressed patients have decreased levels of Glx (a composite measure of glutamate and glutamine) in anterior brain regions (Auer et al., 2000; Hasler et al., 2007; Yildiz-Yesiloglu and Ankerst, 2006). Interestingly a large MRS study of occipital cortex reported an opposite finding, namely that acutely depressed patients had increased glutamate levels (Sanacora et al., 2004), suggesting potential regional brain differences in Glx regulation in depression.

In contrast, there have been few studies of Glx levels in recovered depressed patients but such information is important in determining the pathophysiological role of glutamate in vulnerability to depression. Our group found increased levels of Glx in occipital cortex in recovered unmedicated depressed patients (Bhagwagar et al., 2007), consistent with the increased concentrations of glutamate reported in this brain region in acute depression. However, when we examined Glx levels in a subset of these participants in anterior cingulate cortex we found no difference between patients and controls (Bhagwagar et al., 2008), again suggesting regional differences in Glx regulation in patients with mood disorder. Hasler et al. (2005) also found normal Glx levels in anterior cingulate cortex in recovered depressed participants. The aim of the present study was to examine Glx levels in anterior cingulate cortex in a further group of recovered depressed patients. In addition, because levels of glutamate and glutamine can vary independently of each other we used a further MRS technique, PRESS-J, which permits measurement of glutamate without glutamine at moderate field strengths (Hurd et al., 2004).

## 2 Materials and methods

### 2.1 Participants

Medication-free, fully recovered patients with a history of recurrent major depressive disorder, diagnosed by the Structured Clinical Interview for DSM-IV (*n* = 14; 4M, 10F) and healthy controls (*n* = 16; 5M, 11F) were recruited. All were euthymic for at least three months, and had received no psychotropic medication within three months (mean = 38 months; range 8–84 months); three patients were drug naïve. Nine patients had a family history of mood disorder. Three of the recovered depressed patients had co-morbid illnesses; one had social phobia, and two subjects had a history of alcohol misuse but not dependence. On the day of scanning, mental state ratings were performed with the Hamilton Depression Rating Scale, Beck Depression Inventory, and Spielberger State and Trait Anxiety scales. All participants gave written informed consent to the study, which was approved by the Oxfordshire Research Ethics Committee, UK.

### 2.2 MRS methodology

Scanning was performed on a 3T Varian INOVA system with a head optimised gradient coil (Tesla Engineering, Storrington, UK) and a head-only transmit/receive quadrature birdcage RF coil. Data were acquired from a 30 × 20 × 20 mm voxel placed in medial prefrontal and anterior cingulate cortex anterior to the genu of the corpus callosum. The voxel was positioned manually by reference to an axial T_1_-weighted gradient-echo image with the base of the voxel on a plane running through the genu and splenium of the corpus callosum.

PRESS data with water suppression (TE 26 ms, TR 3 s, 64 averages), and without (TE 26 ms, TR 3 s) were acquired. PRESS-J data (Hurd et al., 2004) with and without water suppression were similarly acquired with TE arrayed from 35 ms to 185 ms in 10 ms increments (water-suppressed data, total acquisitions = 128; non-water suppressed data, total acquisitions = 16; TR = 3 s). T_1_-weighted structural images of whole brain were acquired with 2 mm^3^ voxel resolution.

PRESS data were analysed with LCModel software (Provencher, 1993), using the non-water suppressed data for eddy current correction, calculating metabolite concentrations relative to creatine in conventional fashion using metabolite basis spectra (alanine, aspartate, creatine, GABA, glucose, glutamine, glutamate, glycerophosphocholine, phosphocholine, lactate, myo-inositol, N-acetylaspartate, N-acetylaspartylglutamate, scyllo-inositol, taurine) and simulated lipid and macromolecule components. PRESS-J spectra were zero-order phased, apodised with a 5 Hz Gaussian filter, and summed (Hurd et al., 2004) before analysis. Glutamate levels relative to creatine were measured using AMARES (Advanced method for accurate, robust and efficient spectral fitting) (Vanhamme et al., 1997) since metabolite basis spectra were not available for PRESS-J acquisitions, and the spectral simplification and flat baselines obtained with this technique in vivo make direct single peak fitting reliable (Hurd et al., 2004). Concentrations in proton MRS studies are conventionally expressed relative to creatine which acts as an internal reference standard in the voxel (see for example, Sanacora et al., 2004; Hasler et al., 2005); levels relative to tissue water were also calculated but not included here for reasons of space.

FSL FAST (Zhang et al., 2001) was employed to segment the structural brain images into grey matter, white matter, and CSF, to allow estimation of voxel composition.

### 2.3 Statistical analysis

Statistical analyses were performed in SPSS version 15. To guard against type I error, the available MRS measures (Glx, glutamate, myo-inositol, choline, and N-acetylaspartate) were initially compared between groups by multivariate analysis using Wilks' lambda. Two-tailed univariate analysis for each measure independently was then performed to identify the basis of any overall group effect. The sensitivity of effects to the inclusion or exclusion of covariates (age and voxel composition) was tested. Group demographics and clinical characteristics were compared by *t*-test. Power calculations indicated that a sample size of 30 would have 80% power to detect a 10% difference in Glx/Cr between groups at 0.05 significance level. This is the size of difference typically seen in studies of depressed patients (Auer et al., 2000; Hasler et al., 2007; Yildiz-Yesiloglu and Ankerst, 2006).

## 3 Results

Participants with previous episodes of major depression did not differ significantly from controls in age, or on ratings of current symptoms of depression or anxiety (Table 1). There was a higher level of trait anxiety reported in those with previous depressive illness (36.2 ± 2.6 vs 30.1 ± 1.3, *p* = 0.04).

**Fig. 1:**
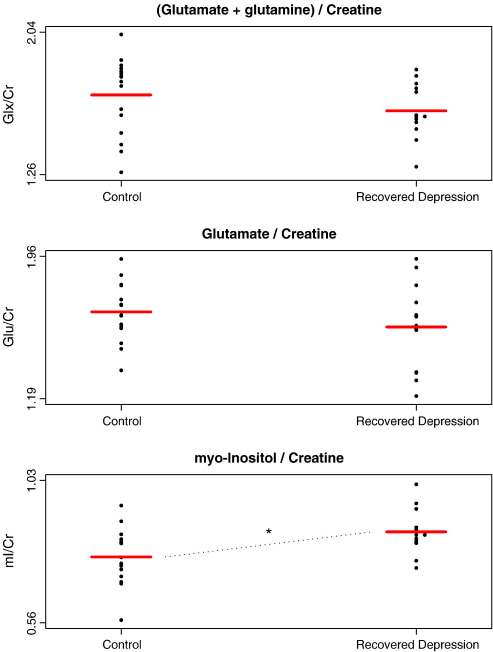
Individual estimates (points) and group means (bars) for levels of glutamate and glutamine (Glx), glutamate, and myo-inositol in healthy controls (*n* = 16) and medication-free patients recovered from depression (*n* = 14). Levels expressed relative to creatine. (⁎, *F*(1,28) = 7.49, *p* < 0.02).

The voxels measured contained predominantly grey matter (69%). Multivariate analysis of MRS results revealed a significant difference between the groups (Wilks' lambda = 0.624, *F*(5,24) = 2.894, *p* = 0.035). However this difference was not explained by glutamatergic abnormalities, since levels of Glx and glutamate did not differ between groups (*p* > 0.2 in both cases; Fig. 1, Table 1). Interestingly, myo-inositol levels were significantly elevated in those with a history of major depressive disorder (*F*(1,28) = 7.49, *p* < 0.02; Fig. 1, Table 1). The elevation of myo-inositol was robust to inclusion of age and voxel composition as covariates (*F*(1,24) = 7.611, *p* < 0.02). The groups did not differ on other MRS measures (NAA, choline, creatine), nor in voxel composition (*p* > 0.1 in each case, Table 1). All findings were robust to the inclusion of voxel grey and white matter composition as covariates.

## 4 Discussion

Our findings suggest that both Glx (glutamate and glutamine) and glutamate alone as measured by proton MRS at 3T are normal in the anterior cingulate cortex of recovered depressed patients withdrawn from medication. Therefore, based on the results of studies in acutely depressed patients, we conclude that the low Glx concentration in anterior brain regions detected by MRS is likely to be a state marker of acute depression. This contrasts with findings in occipital cortex, where levels are apparently elevated in both acutely ill and recovered depressed patients (Bhagwagar et al., 2007; Sanacora et al., 2004).

In some contexts, Glx estimates include a small component (< 10%) attributable to GABA (Sanacora et al., 2008). Reports conflict as to whether reduced levels of GABA are found in the anterior cortex of recovered depressed patients (Bhagwagar et al., 2008; Hasler et al., 2005). In the absence of specific GABA measurements, the data reported here cannot clarify this point since, at most, the reduction in GABA would be reflected by a 1% difference in Glx levels which is beyond the statistical power of this study.

The conclusion that glutamatergic abnormalities in anterior brain regions are markers of the acute depressive state should be treated as tentative until further studies can be performed. For example, the current group of recovered depressed patients was not demonstrated to have lowered glutamate levels in anterior cingulate cortex when acutely depressed. In particular our data cannot exclude the possibility that persistent glutamatergic abnormalities exist in a subgroup of patients. These questions could be clarified by a larger longitudinal study of these measures in depressed patients at different phases of their illness.

MRS metabolite concentrations in this study were expressed relative to creatine, which is conventional when proton MRS is used. However, this is unlikely to have affected the study conclusions since analyses referencing to tissue water found the same pattern of findings (data not shown). It is quite possible that more subtle abnormalities in glutamate mechanisms, for example in glutamate receptor populations could be present in recovered depressed patients but not detectable by MRS (Sanacora et al., 2008). Why recovered depressed patients should apparently have persistent increases in Glx in occipital cortex (Bhagwagar et al., 2007) is not clear at present.

Our finding of elevated levels of myo-inositol in the recovered depressed patients was unexpected. In previous studies of anterior cingulate cortex in acute depression, one study with participants of a similar age range to those described here (Coupland et al., 2005) found decreased myo-inositol, whereas two studies in older populations found no significant difference (Auer et al., 2000; Kumar et al., 2002). Similarly, in studies using voxels placed predominantly in frontal white matter, those with similar age ranges to the current study found lower levels of myo-inositol in acute depression (Frey et al., 1998; Gruber et al., 2003), whereas levels were elevated in a more elderly sample (Kumar et al., 2002). The reason for this apparent effect of age on myo-inositol levels in depression in not known, although it has been hypothesised that it may reflect increased probability of reactive gliosis in more elderly populations (Coupland et al., 2005).

Inositol and related compounds have an important role as intracellular second messengers; however the MRS-visible signal in brain appears predominantly to reflect myo-inositol actively accumulated within astrocytes where it is employed as an osmolyte (Brand et al., 1993). Thus elevated myo-inositol, as well as normalised glutamate and glutamine, after recovery from depression may reflect changes in glial function. Glia plays a key role in the regulation of glutamate neurotransmission though the process of glutamate–glutamine cycling (Sanacora et al., 2008), making it possible that changes in this process during recovery from depression might account for both normalisation of Glx levels and increases in myo-inositol. This hypothesis could be tested by using carbon-13 MRS which permits the measurement not only of overall glutamate levels, but also rates of glutamate–glutamine cycling through glia (Shen et al., 1999).

## Role of funding source

This study was supported by the Medical Research Council, UK. PJC is a MRC Clinical Scientist. MJT was funded by a Wellcome Trust Research Training Fellowship. The Medical Research Council and Wellcome Trust had no further role in study design; in the collection, analysis and interpretation of data; in the writing of the report; and in the decision to submit the paper for publication.

## Conflicts of interest

Dr Cowen has been a member of advisory boards for Eli Lilly, Servier and Wyeth. Prof Jezzard and Drs Taylor, Selvaraj, and Norbury reported no biomedical financial interests or potential conflicts of interest.

## Figures and Tables

**Table 1:** Group characteristics.

	Recovered depression (*n* = 14)	Controls (*n* = 16)
Age (mean and range)	32.6 (18–57)	31.8 (19–63)
Gender	4M, 10F	5M, 11F
Smoking history	5 smokers	3 smokers
Beck Depression Inventory	2.7 (3.1)	1.4 (1.5)
Hamilton Depression Rating Scale	0.3 (0.5)	0.1 (0.5)
Spielberger Trait Anxiety	36.2 (9.7)	30.1 (5.3)⁎
Spielberger State Anxiety	31.5 (8.7)	30.9 (6.2)
Age of onset	22 (10.8)	
Number of episodes	2.6 (1.2)	
Lifetime melancholic depression	3	
Lifetime history of suicide attempts	6	
Months since last antidepressant treatment (mean and range)	38 (8–84)	
Glx/creatine	1.61 (0.15)	1.69 (0.21)
Myo-inositol/creatine	0.86 (0.07)	0.78 (0.09)⁎
N-acetylaspartate/creatine	1.08 (0.09)	1.11 (0.15)
Choline/creatine	0.31 (0.04)	0.29 (0.02)
Glutamate/creatine	1.58 (0.23)	1.66 (0.16)

Mean values with standard deviation unless otherwise stated.

⁎ *p* < 0.05 *t*-test.
